# Assessing the Food and Drug Administration’s Risk-Based Framework for Software Precertification With Top Health Apps in the United States: Quality Improvement Study

**DOI:** 10.2196/20482

**Published:** 2020-10-26

**Authors:** Noy Alon, Ariel Dora Stern, John Torous

**Affiliations:** 1 Beth Israel Deaconess Medical Center Boston, MA United States; 2 Harvard Business School Boston, MA United States

**Keywords:** mobile health, smartphone, Food and Drug Administration, software, mobile phone

## Abstract

**Background:**

As the development of mobile health apps continues to accelerate, the need to implement a framework that can standardize the categorization of these apps to allow for efficient yet robust regulation is growing. However, regulators and researchers are faced with numerous challenges, as apps have a wide variety of features, constant updates, and fluid use cases for consumers. As past regulatory efforts have failed to match the rapid innovation of these apps, the US Food and Drug Administration (FDA) has proposed that the Software Precertification (Pre-Cert) Program and a new risk-based framework could be the solution.

**Objective:**

This study aims to determine whether the risk-based framework proposed by the FDA’s Pre-Cert Program could standardize categorization of top health apps in the United States.

**Methods:**

In this quality improvement study during summer 2019, the top 10 apps for 6 disease conditions (addiction, anxiety, depression, diabetes, high blood pressure, and schizophrenia) in Apple iTunes and Android Google Play Store in the United States were classified using the FDA’s risk-based framework. Data on the presence of well-defined app features, user engagement methods, popularity metrics, medical claims, and scientific backing were collected.

**Results:**

The FDA’s risk-based framework classifies an app’s risk by the disease condition it targets and what information that app provides. Of the 120 apps tested, 95 apps were categorized as targeting a nonserious health condition, whereas only 7 were categorized as targeting a serious condition and 18 were categorized as targeting a critical condition. As the majority of apps targeted a nonserious condition, their risk categorization was largely determined by the information they provided. The apps that were assessed as not requiring FDA review were more likely to be associated with the integration of external devices than those assessed as requiring FDA review (15/58, 26% vs 5/62, 8%; *P*=.03) and health information collection (24/58, 41% vs 9/62, 15%; *P*=.008). Apps exempt from the review were less likely to offer health information (25/58, 43% vs 45/62, 72%; *P*<.001), to connect users with professional care (7/58, 12% vs 14/62, 23%; *P*=.04), and to include an intervention (8/58, 14% vs 35/62, 55%; *P*<.001).

**Conclusions:**

The FDA’s risk-based framework has the potential to improve the efficiency of the regulatory review process for health apps. However, we were unable to identify a standard measure that differentiated apps requiring regulatory review from those that would not. Apps exempt from the review also carried concerns regarding privacy and data security. Before the framework is used to assess the need for a formal review of digital health tools, further research and regulatory guidance are needed to ensure that the Pre-Cert Program operates in the greatest interest of public health.

## Introduction

### Background

The development of mobile health apps has been increasing in recent years; recent estimates found that approximately 325,000 mobile health apps are available in the marketplace [1]. However, a consequence of rapid technological development is that many health apps remain to be unevaluated by researchers [2]. Thus, clinicians and patients are largely uninformed about the efficacy of these apps and lack data on their potential to benefit health and/or cause harm.

Despite a lack of evidence and in the absence of direct regulation, smartphone ownership and interest in health apps remain to be high among patients [3-5] and those who might not have a diagnosis but are seeking to improve their well-being [6-10]. However, the majority of the population has still not downloaded health apps [3,9-11], and clinicians are hesitant to recommend apps [12] because of concerns over privacy, data security [6-9,11], and app effectiveness [7,12]. As such, the need for evidence, guidance, and thoughtful regulation in digital health is clear [13]. More concrete government regulations have the potential to set a quality baseline and reduce the number of unsubstantiated claims made by health apps. These measures could increase clinicians’ and patients’ trust in digital health tools [14].

In the past, the US Food and Drug Administration (FDA) focused its regulatory efforts on a small subset of mobile medical apps: those that provided treatment or diagnosis to users and those that were an extension of or transformed into regulated medical devices [15]. These mobile medical apps would be subjected to a formal FDA review and the same regulatory requirements as other medical devices [15]. This review process requires app developers to register their organization and product and provide information regarding the design procedure, facilities, and how their app will be described. Depending on the classification of their product, developers must also submit a premarket notification or approval with supporting clinical evidence [16]. However, the FDA has acknowledged that this framework is not well suited for rapid development and changes made to many health apps [17].

### The Software Precertification Pilot Program

As a result, in June 2018, the FDA published a working model for its Software Precertification (Pre-Cert) Pilot Program and released a *Test Plan* for the program in early 2019 [18,19]. This program hopes to provide a more efficient review process for software-only products that would reduce the regulatory burden of entering certain software product markets and would encourage software developers to advance the capabilities of their products [20]. The Pre-Cert Program is designed to address many of the tensions between software development and traditional regulated medical technologies [17,18], such as the tradition of regular product updating (by software developers) versus testing and quality assurance before infrequent and discrete product updates (by medical device developers) [21,22]. The FDA’s work on piloting the Pre-Cert Program has continued in recent months, with ongoing evaluation and mid-2019 reporting on how mock reassessments of already-approved software products would have fared under a streamlined regulatory review process [23].

Under the Pre-Cert Program, FDA regulators plan to first evaluate digital health app developers and not the apps themselves [17]. In its current form, the program will only apply to developers marketing software as a medical device (SaMD), which the FDA defines as software that is intended to be used for medical purposes without a hardware extension [24]. Medical purposes applicable to SaMD are defined by the FDA as including but not limited to the diagnosis, prevention, monitoring, treatment, and alleviation of disease or injury [25].

**Figure 1:**
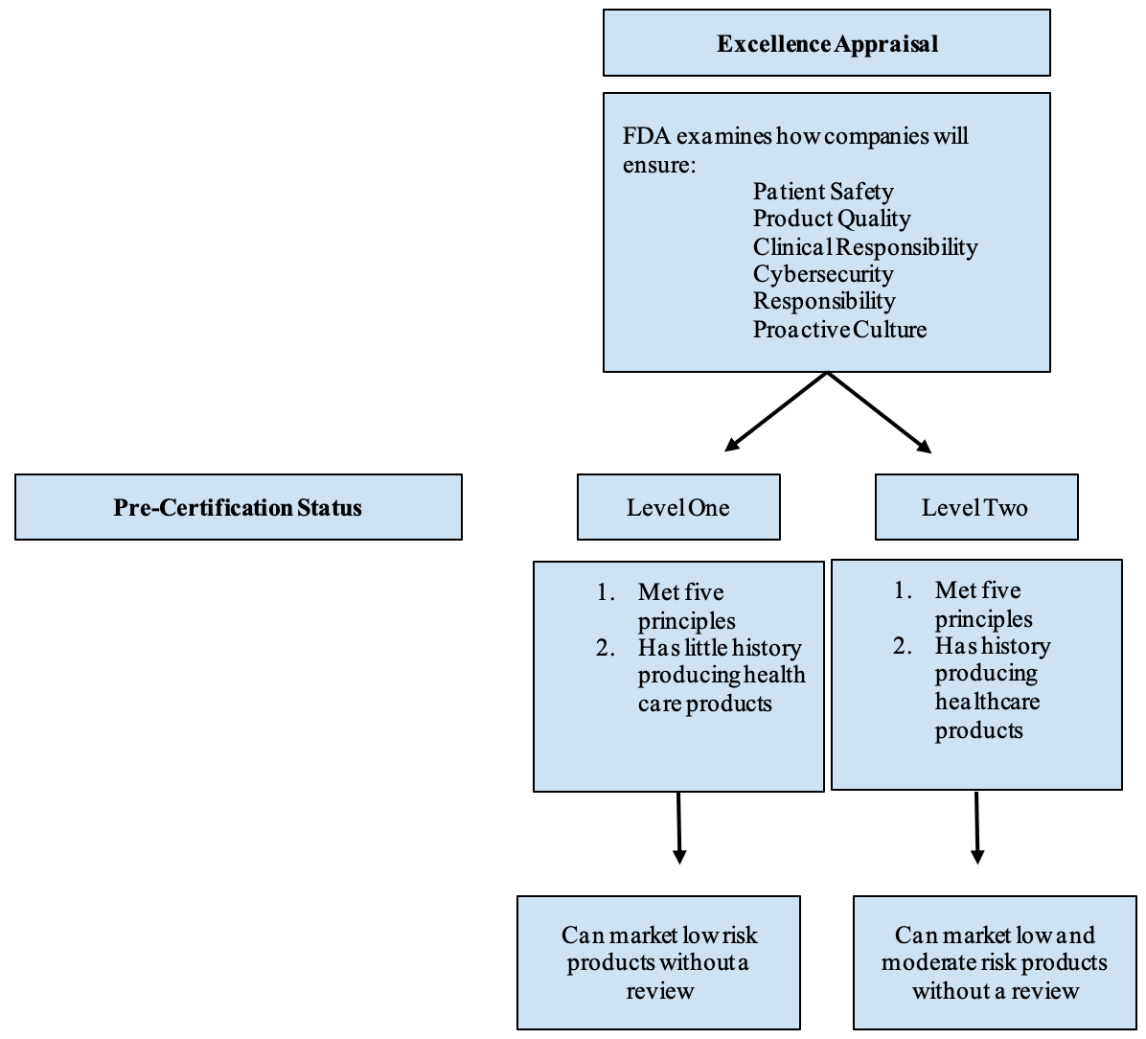
Precertification status determination process. This figure is an overview of how the FDA will determine the precertification status of different organizations. FDA: Food and Drug Administration.

Under Pre-Cert, FDA regulators would first examine companies through an *excellence appraisal*, during which the FDA would review app developers’ policies and practices to determine if and how a developer’s policies enable the organization to excel in 5 proposed excellence principles: (1) patient safety, (2) product quality, (3) clinical responsibility, (4) cybersecurity responsibility, and (5) a proactive culture [18]. If a developer is deemed to have met all 5 principles, the FDA will grant it 1 of 2 precertification statuses: A *Level One or Level Two Pre-Cert.* A *Level One Pre-Cert* would enable organizations to market lower-risk software products without any regulatory review, but moderate- and high-risk products would receive the benefit of undergoing a *streamlined* (abbreviated) review process. This status will be given to developer companies that have met the 5 principles but have less experience producing health care products. A *Level Two Pre-Cert* would enable developer companies to market low- and moderate-risk products without any regulatory review (but would require review for high-risk products) and would only be rewarded to developers that both excelled in the 5 principles *and* have a history of producing safe and effective health care products [18]. This process is shown in Figure 1.

After a developer’s Pre-Cert status is granted, the type of review (if any review is necessary) that each new software product will undergo would be determined by its risk profile. In addition to the developer’s Pre-Cert status, each new software must complete a risk analysis, and together, these designations will determine if a review is necessary. Using a risk-based framework developed by the International Medical Device Regulators Forum (IMDRF) SaMD Working Group, software developers will perform this risk analysis and determine an SaMD’s risk by considering the severity of the medical condition it targets and the type of information the app offers [26]. The IMDRF framework categorized medical conditions as nonserious, serious, or critical, and the FDA has further specified the characteristics of each categorization for the Pre-Cert Program. Similarly, the IMDRF broke down the significance of app-provided information into informing clinical care, driving clinical management, or treating and diagnosing, and the FDA uses these categories in the Pre-Cert Program [18]. The combination of these two-dimensional categorizations, coupled with an organization’s Pre-Cert status, will then jointly determine whether the FDA would perform a regulatory review for a given SaMD product. If necessary, a review would then be completed before an SaMD product can be marketed. Importantly, the FDA plans to continue regulating products that come to market through the Pre-Cert process by continuously examining their *real-world performance* in the postmarket setting [18,27].

The Pre-Cert Program hopes to streamline the FDA’s review process by incorporating FDA oversight during the development of precertified organizations’ apps and not just when the app is finalized. The FDA also hopes to minimize the burden on developers to prove their product’s efficacy and safety, but the list of reduced requirements has yet to be finalized [17].

### Current Frameworks

The FDA’s effort to modernize its regulatory framework is not unique, as multiple guidelines attempting to clarify and streamline government regulations of digital health tools have been implemented in both the United States and Europe. In conjunction with the FDA and other departments, the Federal Trade Commission has developed a web-based survey helping app developers identify what federal regulations pertain to their app [28]. In Europe, the European Commission eHealth Action Plan was first adopted in 2004 and has worked to clarify policies and present the possibilities of using digital health tools to populations throughout the European Union [29]. In 2017, the World Health Organization published guidelines for classifying digital health interventions [30]. In March 2019, the UK National Institute for Health and Care Excellence published a framework establishing standards of evidence for these technologies [31].

In addition, the clinical community has already begun the process of evaluating apps (including highlighting concerns around both patient privacy and app efficacy) [32-35] and thus has gained insight into how patients use apps and the current quality of available apps. As the clinical community has gained experience with digital health and patients and clinicians will be the ones using and recommending these tools, we sought to determine if their findings and concerns are reflected in this new model. To achieve this goal, we simulated the Pre-Cert Program’s risk categorization of SaMD products and considered whether such a risk-based framework would be able to differentiate top health apps based on their features. We aimed to determine if there is a correlation between apps’ features, attributes, and functionality and their Pre-Cert risk categorization. This correlation could indicate that the framework is reliable and accurate and thus would provide a standardized way to characterize these technologies.

## Methods

### Data Collection

We based our analysis on the methods outlined in a study by Wisniewski et al [32] following published and peer-reviewed methods for app selection and classification that attempt to standardize app categorization. Patient feedback was involved in the design of the codebook used in the study, determining what app features should be included. On June 20, 2019, the top 10 apps in both Apple iTunes and Android Google Play Store in the United States were selected out of a total of 120 apps for 6 common disease conditions: addiction, anxiety, depression, diabetes, high blood pressure, and schizophrenia. As 10 apps for each disease condition were chosen across 2 platforms, 20 apps in total were assessed for each condition. The top 10 apps represent the sample that consumers would likely be exposed to first and thus, most likely to use. As a result, although this sample is a convenience sample, it has clinical and real-world relevance. As the methodology was based on Wisniewski et al [32], the same disease conditions were studied to maintain consistency. In total, 2 independent coders downloaded, used, and evaluated the apps. Dummy profiles were created, and each app was tested with data provided by the researchers. Any disagreements between coders were resolved via discussion until a consensus was reached. All the raw data and significance testing performed were reviewed by a clinician who offered guidance on the clinical relevance of the data. Each coder completed a spreadsheet that prompted information identical to that of Wisniewski et al [32], including the presence of app attributes (such as a written privacy policy), information on how the app gathered and returned data, stated patient engagement methods, visible popularity metrics, stated medical claims, and the presence (or absence) of scientific evidence (ie, evidence-based claims). Wisniewski et al [32] is useful for further reference. Only the presence of observable features was coded for each app. Features such as *ease of use* are dependent on the user and were not included in the codebook in an attempt to achieve reliability between coders in an app’s categorization. A risk-based framework, following the characteristics outlined in the FDA’s current draft of the Pre-Cert Program, was added to the coding procedure to simulate applying the model in potential regulatory use [18]. A correlation between the presence of apps’ features, attributes, and functionality and their Pre-Cert risk categorization could point to the framework’s reliability and accuracy when categorizing apps. For example, if an app’s ability to access the phone’s camera is found to differentiate between apps that require a review and those that are exempt, then this result suggests that the Pre-Cert’s risk categorization is not based on subjective measures and has the potential to weed out the apps that pose a greater risk to consumers. Thus, both metrics were coded for, and comparisons were made between them.

**Figure 2:**
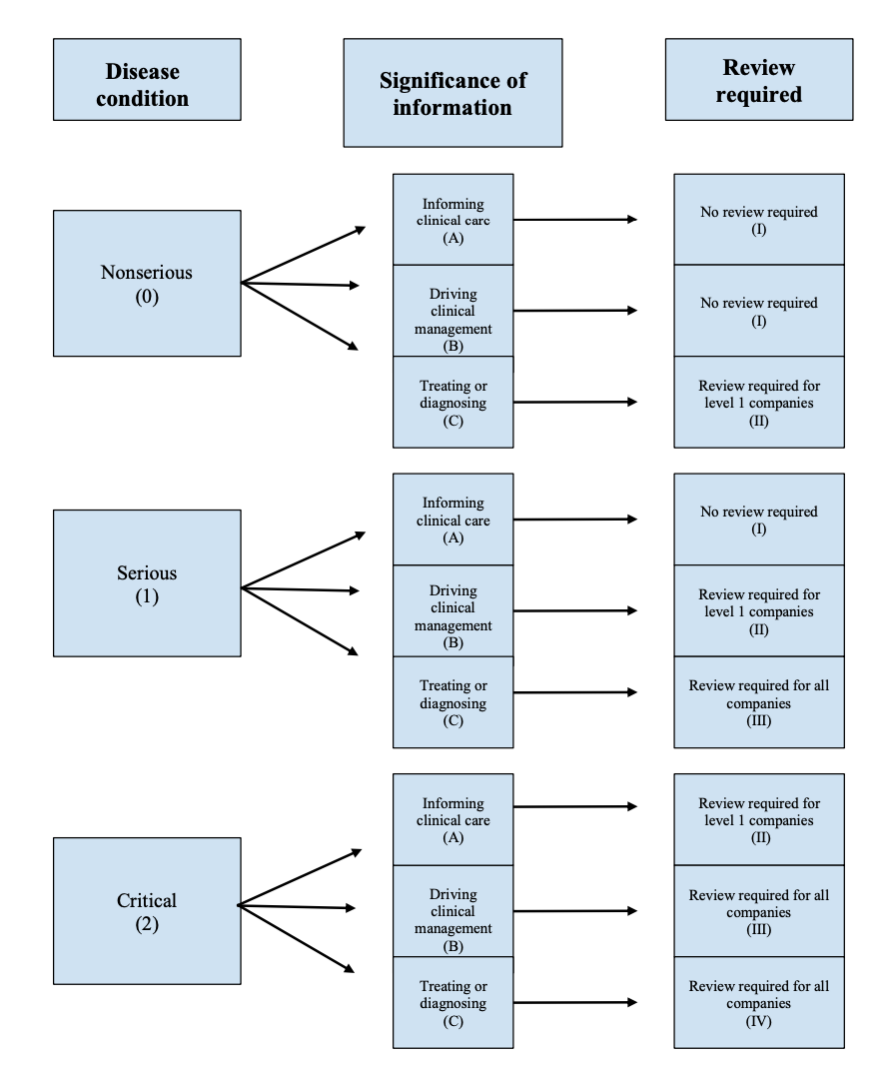
Risk categorization rating system. This figure shows how the Pre-Cert Program uses the disease condition an app targets (0-2) and what information that app provides (A-C) to determine what review that app must undergo (I-IV).

We translated both the disease condition and significance of information categories to a numerical scale, allowing for easier data analysis: apps that were deemed to target a nonserious condition were rated as 0, whereas serious and critical conditions were given a 1 and 2, respectively. If an app targeted several diagnoses, it was categorized by the most severe disease condition described. Regarding the information provided, informing clinical care was rated an “A,” whereas apps that drove clinical management or treated and diagnosed users were given a “B” and “C,” respectively. Using the FDA’s current guidelines, we coded apps as informing clinical care if they simply provided information. Any personal data entry that was used to monitor symptoms was coded as driving clinical management, whereas apps providing treatment and diagnosis were differentiated from other functionalities. An app’s review required status, the classification that decides whether an FDA review would be required under the Pre-Cert Program, was determined by the combination of both criteria and given the numerical value that the IMDRF working group had previously attributed to each category, ranging from 1 to 4 (I, II, III, and IV). For example, a meditation app claiming to alleviate anxiety and stress would have been coded as targeting a nonserious condition (0) and providing treatment (C). Under the Pre-Cert Program, this app would be given a review level of II, which requires *Level One Pre-Cert* organizations to undergo a streamlined review, whereas *Level Two* organizations are exempt from any FDA review. The rating system used is shown in Figure 2. Following the previous literature, we coded only for the presence of and not the quality of app features to maintain the reliability of the data.

### Statistical Analysis

Following coding and data reconciliation, apps were dichotomized into exemption from a review or requiring a review. Apps given an IMDRF categorization of “I” would be exempt from any regulatory review, whereas type II, III, and IV apps would undergo some form of review depending on the precertification status of the organization. As types II, III, and IV apps would undergo some form of review, we grouped these apps together. The data were further stratified by categorical measures, such as which disease condition they targeted. Two-sided *t* tests of differences between categories under the assumption of equal variances were performed using Microsoft Excel 2019 (version 16.0.6742.2048) to determine statistical significance.

## Results

### Principal Results

Of the 120 total Apple and Android apps examined in the simulation, 95 (79.2%) were categorized as targeting a nonserious health condition, whereas only 7 (5.8%) apps targeted a serious condition, including one app that targeted addiction, 5 that targeted depression, and one that targeted anxiety. The remaining 18 (15.0%) apps targeted a critical condition; however, all apps in this group targeted schizophrenia.

Review required status—that is, the classification that determines if an FDA review would be required under the Pre-Cert Program and represented by code I, II, III, or IV (Figure 2)—was largely determined by the information that the app’s developer provided. Of 120 apps, 30 (25.0%) were found to have informed clinical care, whereas driving clinical management and treating or diagnosing each had 42 (35.0%) apps fitting their respective descriptions. The significance of information, which is coded A, B, or C (Figure 2), for the remaining 6 (5.0%) apps was unclassifiable, as these apps were not intended for or did not claim to provide any health-related advice or treatment. As these apps did not offer any health information, they were deemed to be exempt from an FDA regulatory review. These apps were included in the original sample as a general search in both app stores was performed in an attempt to mimic the experience of consumers if they searched for health apps. As a result, not all apps were necessarily marketed under the store’s medical category.

When comparing the reliability between Apple and Android apps, no statistically significant differences were found between whether or not review was required for each disease condition between the platforms (addiction: 1.25 vs 1.22, *P*=.72; anxiety: 1.8 vs 2, *P*=.34; depression: 1.8 vs 2, *P*=.55; high blood pressure: 1.22 vs 1.3, *P*=.41; and schizophrenia: 2.22 vs 2.56; *P*=.57). This suggests that the Pre-Cert’s categorization is reliable across platforms. As both platforms are available for consumers to choose from, it is worthwhile to note the lack of statistical significance between them.

### Stratification by Review Required Status

**Figure 3:**
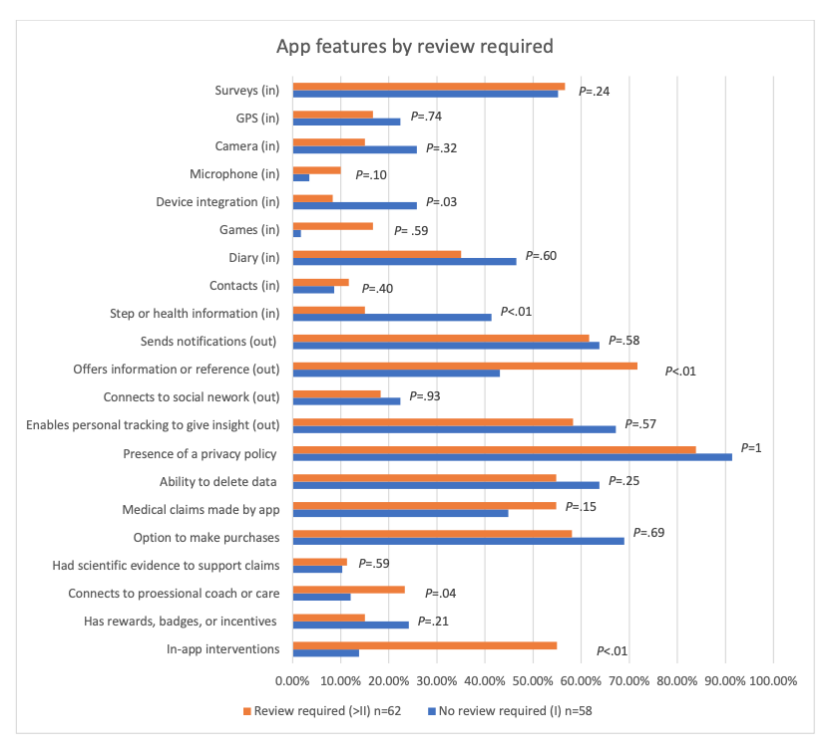
App features by review required. The orange bars represent apps that would undergo a regulatory review in the Pre-Cert Program (review levels II, III, and IV), and the blue bars represent apps exempt from review (review level I).

The number of apps and their features were stratified by whether a review was required and are shown in Figure 3. The features coded for are grouped thematically, as features associated with gathering data are labeled (in) and those involved with user engagement or presenting information are labeled (out; Figure 3). App attributes associated with privacy, medical claims, presence of scientific evidence, connection to professional care, and use of rewards or inventions are listed below.

Two-sided *t* tests comparing all app features described between apps that the Pre-Cert Program exempted from review and those that would require a regulatory review were performed (Figure 3). Apps with a review level of 2 or greater were combined because of the small number of apps under the third and fourth review levels (III and IV) and because all these apps would require a review. Apps that did not require any review (review level I) were more likely than those that definitely or may have required review to integrate data from external devices such as smartwatches (15/58, 26% of review level I apps vs 5/62, 8% of reviews II, III, *and IV level apps*; *P*=.03) and collect health information such as step counts (24/58, 41% vs 9/62, 15%; *P*=.008). Apps expected to be exempt from FDA review (I) were less likely to offer information or reference facts (25/58, 43% vs 45/62, 72%; *P*<.001), were less likely to connect to professional care (7/58, 12% vs 14/62, 23%; *P*=.04), and were less likely to include an intervention (8/58, 14% vs 35/62, 55%; *P*<.001) than those requiring a review. User-given star ratings also significantly differed between apps that did not require review versus those that definitely or may have required review (4.48 vs 4.13; *P*=.003), suggesting that streamlined apps were rated more highly than those definitely or potentially requiring a review. Notably, there were no statistically significant differences between the 2 potential review profiles in the provision of information about the ability for data deletion (37/58, 64% vs 34/62, 55%; *P*=.20) or the average days since the app’s last update (189 vs 264; *P*=.60).

The mean values and SDs of proxies for app popularity (star ratings, number of reviews, and days since the last update) and data on days since each app’s last update are summarized in Table 1.

**Table 1:** Popularity metrics and update history by review required.

Review required	No review required (n=58)	Review required (n=62)
User star ratings, mean (SD)	4.48 (0.6)	4.13 (1)
Number of ratings, mean (SD)	14,554 (42,409)	14,018 (70,454)
Days since last update, mean (SD)	189 (335)	264 (338)

### Stratification by Targeted Disease

When the data were stratified by targeted disease, the number of apps requiring review within each condition varied dramatically. In total, 16 apps targeting addiction required no review (I), while only 4 apps would undergo a review (II, III, and IV). A similar trend is seen in apps targeting high blood pressure as 15 of these apps were determined to be exempt from a review (I), leaving only 5 apps to undergo a review (II, III, and IV). Notably, all apps targeting diabetes were deemed to be exempt from any review (I), whereas anxiety, depression, and schizophrenia apps comprised the majority of those definitely or possibly requiring review (II, III, and IV). This result was driven by the finding that most anxiety and depression apps offered treatment and schizophrenia was classified as a critical disease condition, which resulted in a higher likelihood that review would be required.

When stratified by disease, the samples become underpowered because of the small number of apps in each subgroup. However, it is worth noting that some of the differences noted earlier remain. For example, only 6% (1/16) of addiction apps exempt from review offered an intervention, whereas 100% (4/4) of addiction apps requiring a review did. The same trend holds true for apps targeting anxiety (0/3, 0% vs 16/17, 94%) and depression (1/6, 17% vs 11/14, 79%). In addition, none of the apps targeting depression that were exempt from review offered external information, whereas 79% (11/14) of those requiring reviews did. Notable results are summarized in Tables 2 and 3, whereas a full table comparing app features and whether review would be required, stratified by targeted disease, is provided in Multimedia Appendix 1.

**Table 2:** Apps’ features for addiction, anxiety, and depression by review required, stratified by targeted disease.

App features	Addiction apps exempt from review (n=16)	Addiction apps requiring reviews (n=4)	Anxiety apps exempt from review (n=3)	Anxiety apps requiring reviews (n=17)	Depression apps exempt from review (n=6)	Depression apps requiring reviews (n=14)
Device integration, n (%)	0 (0)	0 (0)	0 (0)	2 (12)	1 (17)	0 (0)
Steps or health information, n (%)	0 (0)	0 (0)	1 (33)	3 (18)	1 (17)	3 (21)
Offer information, n (%)	2 (13)	4 (100)	2 (67)	12 (71)	0 (0)	11 (79)
Connect to professional care, n (%)	5 (31)	1 (25)	1 (33)	6 (35)	0 (0)	6 (43)
In-app interventions, n (%)	1 (6)	4 (100)	0 (0)	16 (94)	1 (17)	11 (79)
User star ratings, mean (SD)	4.7 (0.18)	4.68 (0.17)	4.5 (0.52)	4.65 (0.19)	4.55 (0.23)	4.2 (0.56)

**Table 3:** Apps’ features for diabetes, high blood pressure, and schizophrenia.

App features	Diabetes apps exempt from review (n=20)	High blood pressure apps exempt from review (n=15)	High blood pressure apps requiring review (n=5)	Schizophrenia apps exempt from review (n=4)	Schizophrenia apps requiring review (n=16)
Device integration, n (%)	11 (55)	3 (20)	0 (0)	0 (0)	3 (19)
Steps or health information, n (%)	8 (40)	1 (7)	0 (0)	0 (0)	3 (19)
Offer information, n (%)	14 (70)	5 (33)	1 (20)	2 (50)	15 (94)
Connect to professional care, n (%)	1 (5)	0 (0)	0 (0)	0 (0)	1 (6)
In-app interventions, n (%)	6 (30)	0 (0)	1 (20)	0 (0)	1 (6)
User star ratings, mean (SD)	4.48 (0.47)	4.13 (0.94)	3.28 (1.19)	4.18 (0.57)	2.5 (1.9)

## Discussion

### Principal Findings

After coding for the presence of observable features of top health apps, we found attributes that differentiated the apps that would likely undergo an FDA regulatory review under the Pre-Cert Program versus those that would not. Apps offering interventions were most likely to require a review (II, III, and IV), whereas monitoring apps were more likely to be streamlined. In addition, apps requiring FDA review were more likely to offer references and connect users to professional care than streamlined apps. This distinction between formal medical advice and user-led data embedded in the FDA’s risk categorization demonstrates a promising foundation for the framework. Apps gearing themselves toward providing more formal care, such as interventions or references, have the potential to elicit greater harm than monitoring apps if these features are erroneous. Consumers are using these apps for treatment or diagnosis and are being exposed to the information provided by these health apps. Monitoring apps largely rely on data provided by the user rather than on the supply of novel information. The Pre-Cert’s risk categorization’s ability to differentiate between apps relying on formal medical advice versus user-led data and require a review from the former indicates its potential to catch apps that pose a greater risk.

When the data were stratified by targeted disease, the sample became underpowered, and we were unable to perform significance testing. However, although the small size of each subgroup is a limitation in this study, the observed trends offer valuable and novel insight into how the Pre-Cert Program’s categorization should be refined before full implementation. For example, in the subgroup analysis, the percentage of apps offering an intervention differed dramatically between those exempt from review and those requiring a review for apps targeting addiction, anxiety, and depression. This finding hints that the presence of an intervention is one of the strongest associations for apps requiring a review. However, this metric is challenging to reliably differentiate from apps that simply monitor symptoms. In particular, in the mental health setting, it has been established that individuals who monitor their symptoms feel better [36,37], and the line between ecological momentary assessments versus intervention is blurred with apps. Determining whether an app drives clinical management or provides treatment is pivotal for that app’s risk categorization under Pre-Cert. Therefore, the FDA should set clear guidelines and give examples of what apps they consider provide an intervention versus monitor symptoms. Although we coded 24 features of apps (Figure 3) and (Table 1), only a few were found to differentiate between apps that would require FDA review and those that would have been streamlined. In addition, as described earlier, the strongest association between apps requiring a review that are offering an intervention remains to be difficult to clearly define. These results suggest that if the FDA wants to implement an appropriately detailed risk-based framework that can address the features of already-existing apps, they will need to publish more explicit guidelines and likely require extensive training for coders in order to obtain high interrater reliability and avoid possible misclassifications.

A specific criterion that the FDA should set more explicit guidelines around is the disclosure of apps’ data policies. At present, the framework does not reflect if or how an app discloses how users can delete their information. For example, only 64% (37/58) of apps evaluated as likely to be exempt from review provided information about data deletion, although *cybersecurity responsibility* remains one of the Pre-Cert Program’s 5 excellence principles. Apps requiring review levels of 2 or higher had a similar percentage of 55% (34/62). This result indicates that apps containing features and functions that pose legitimate risks on user privacy and data security are exempt from review under the Pre-Cert Program. Patients and clinicians who today use medical apps note *privacy and security* to be one of their top concerns with mobile health, meaning there is an opportunity for the FDA to be more explicit when assessing app developers’ data policies.

The Pre-Cert Program and its risk categorization are still in their early developmental stages, as the FDA continues to test myriad aspects of the program. In an update summarizing testing performed through May 2019, the FDA described their refinement of this review determination process and admitted that further insight from patients and the digital health community is needed [23]. Our results can help inform the program in these pivotal early stages. To make the framework more useful, more data concerning its strengths and weaknesses are necessary. A novel challenge for evaluating apps is that their use case is not static and will vary based on the patients’ clinical needs and treatment goals. For example, a mindfulness app may be a well-being tool, exempt from regulation in some contexts, but could also be recommended by clinicians in the treatment of major depressive disorders. This fluidity of purposes is different from that of a traditional medical device, for example, a pacemaker, which has a narrow and well-defined use among patients. In addition, further clarification as to what the FDA defines as a nonserious, serious, and critical condition and what regulators consider to be informing clinical care, driving management, or treating and diagnosing should be published. Finally, as the FDA plans to surveil an app’s *real-world performance* in a postmarket setting, it will be important for them to publish guidelines of how they will ensure that not only the app’s quality remains consistent but also that the developer continues to excel in the 5 criteria that granted them Pre-Cert status initially. Creating optimal systems is complex and will require the right combination of diverse stakeholder involvement. Thus, clinicians, patients, and leaders in the digital health community should be fully incorporated into this process and will likely welcome the opportunity to provide feedback.

### Limitations

Our results must be interpreted in light of several limitations. First, we examined only 120 apps out of thousands that are currently marketed. As we took a convenience sample, there is the possibility that this sample does not reflect the top 10 apps presented to every consumer upon their search. In addition, at present, it is unclear which apps will need to be regulated with Pre-Cert and which will voluntarily partake—although as many health-related apps at present make clinical claims, many likely would fall under the scope of regulation. Second, our ratings were obtained by 2 reviewers and checked by a third reviewer. No third-party standards currently exist for determining how to score apps and to maintain validity, although we used published evaluation standards from previous research. Our research team only coded those features that could be verified, meaning that more subjective aspects of software products, such as app usability, were not coded. Third, we recognize that apps targeting certain disease conditions will have some inherent features and classifications. For example, apps targeting diabetes are more likely to integrate external devices than apps targeting other disease conditions because of their connection to blood glucose monitors. We attempted to minimize this effect by having a large sample of apps across a diverse range of conditions. In addition, the Pre-Cert’s risk categorization will be used to classify these mobile health apps and will also need to account for these inherent features. Finally, we acknowledge that this risk-based framework is still in its testing phase and that revisions and additions will be made that are likely to increase the clarity of the criteria. Indeed, we expect this and applaud ongoing FDA efforts to pilot its framework and invite feedback from user communities.

### Comparison With Prior Work

In the current state of digital health, the need to provide these more explicit guidelines is clear. There remains a lack of standards-based and reliable regulatory frameworks and evaluations that assess app quality, which, in turn, diminish consumers’ confidence in digital health [38]. Researchers have attempted to bridge this gap by testing the reliability and accuracy of current rating systems [13,33,35] and deriving their own frameworks [34,38]. Our study fits into this landscape as we simulated using a proposed framework, the Pre-Cert Program’s risk categorization, to classify top health apps. We provide novel information, as the Pre-Cert’s framework has not been used on a sample of currently available apps. Thus, our study identifies the potential of the program and areas for its improvement, which can inform other app classification initiatives. In addition, our studies and others similar to ours could potentially be used to surveil apps after they have reached the market. We used a peer-reviewed and published method that standardizes app selection and classifies apps based on 24 features. The iterations of this study over time can track if an app updates its features or changes its policies. Therefore, the community could play a role in the postmarket surveillance that FDA plans to implement in the Pre-Cert Program. We were limited by the fact that this risk categorization has not been previously studied as other rating systems. The lack of guidance and clarifications when discrepancies arose is an area of concern as digital health remains to be an evolving and sometimes ambiguous landscape.

### Conclusions

The Pre-Cert Program’s risk-based framework for assessing digital health apps and other SaMD products offers a promising foundation for enforcing appropriate digital health regulation while facilitating innovation and the use of technological advancements. However, the limited differences in our sample between apps likely requiring regulatory review and those that likely do not suggest that more detailed criteria are needed. We believe that additional exercises such as those done in this study, which can shed light on how the framework is likely to play out in the context of real-world digital health products, will be of high value. On the basis of such research, regulatory guidelines could be clarified and specified before the framework is deployed in the complex and dynamic landscape of digital health.

## Appendix

Multimedia Appendix 1 Apps reviewed.

## Abbreviations

FDA: Food and Drug Administration
IMDRF: International Medical Device Regulators Forum
Pre-Cert: Software Precertification
SaMD: Software as a Medical Device
