# Supplementary material for: Assessing the Food and Drug Administration’s Risk-Based Framework for Software Precertification With Top Health Apps in the United States: Quality Improvement Study
Source: JMIR Mhealth Uhealth. 2020 Oct 26;8(10):e20482. doi: 10.2196/20482 (PMC7652687; doi:10.2196/20482)
Supplement: Multimedia Appendix 1 [file mhealth_v8i10e20482_app1.docx]

| **Apps Reviewed** | |
| --- | --- |
| **Apple** | **Android** |
|  |  |
| **Addiction** | |
| I am Sober | Sobriety Counter - Bad Habits |
| Nomo - Sobriety Clocks | I Am Sober |
| Addiction Solitaire | Addiction Solitaire |
| Brainbuddy: Quit Porn Forever | Pocket Rehab: Get Sober & Addiction Recovery |
| Quit Addiction Buddy: Recovery to Super Power | Sober Time - Sober Day Counter & Clean Time Clock |
| Quit That! - Habit Tracker | SoberTool - Alcoholism, Addiction, Sobriety Help |
| Fortify - Quit Porn for Good | 12 Step Guide Narcotics Addicts |
| Twenty-Four Hours a Day | No More! Quit your Addictions |
| Sober Time - Sobriety Counter | Addiction Quotes |
| Add-Iction | NA Speaker Tapes & Workshops Addiction Recovery |
| **Anxiety** | |
| Pacifica for Stress & Anxiety | Pacifica - Stress & Anxiety |
| AntiStress Anxiety Relief Game | Anxiety Release based on EMDR |
| Daylio Journal | Rootd - Panic Attack & Anxiety Relief |
| Moodpath: Depression & Anxiety | Youper - Emotional Health |
| My Oasis: Relaxing Clicker App | Wysa: Stress, Depression & Anxiety Therapy Chatbot |
| Headspace: Meditation & Sleep | InnerHour- Self Help for Anxiety & Depression |
| Aura: Sleep & Mindfulness | Dare - Break Free From Anxiety |
| Instant Heart Rate: HR Monitor | Moodpath - Depression & Anxiety Test |
| Jour - Guided Journaling | Stress & Anxiety Companion |
| Super Slime Simulator | Anxiety Tracker - Stress and Anxiety Log |
| **Depression** | |
| Moodpath: Depression & Anxiety | Moodpath - Depression & Anxiety Test |
| Depression Test | Youper - Emotional Health |
| Replika | UP! Depression, Bipolar & Borderline Management |
| Youper | Wysa: Stress, Depression & Anxiety Therapy Chatbot |
| Motivation Quotes- Daily Quote | Depression Quote Wallpapers |
| AntiStress Anxiety Relief Game | MoodTools - Depression Aid |
| StressScan - check your stress | Depression Test |
| Reflectly | Depression Test |
| Pacifica for Stress & Anxiety | Depression Quotes |
| Mood - Journal & Anxiety Chat | Mood Tracker & Questions Diary (Anti Depression) |
| **Diabetes** | |
| Glucose Buddy Diabetes Tracker | Diabetes & Diet Tracker |
| Carb Manager: Keto Diet App | mySugr - Diabetes App & Blood Sugar Tracker |
| Sugar Sense Diabetes App | Diabetes: M |
| Diabetes Recipe App | Blood Sugar Log - Diabetes Tracker |
| mySugr - Diabetes Tracker Log | Beat Diabetes |
| ADA Standards of Care | Type 2 Diabetes Healthy Eating |
| Glucose - Blood Sugar Tracker | Diabetes diary - Log Blood Sugar Levels |
| Diabetes Tracker by MyNetDiary | All About Diabetes - A Complete Diabetes Guide |
| OneTouch Reveal | Diabetic Recipes: Great Recipes for Diabetics |
| Noom | American Diabetes Association Standards of Care |
| **High Blood Pressure** | |
| Blood Pressure Companion | Blood Pressure Checker Diary: BP info History Log |
| Finger Blood Pressure Calculator Prank | Lower Blood Pressure Foods |
| Blood Pressure Monitor Log | Blood Pressure Diary |
| High Blood Pressure | High BP Hypertension Diet High Blood Pressure Help |
| Blood Pressure Lite - BP Tracker for Hypertension Management | Blood Pressure Evaluation |
| Health + Blood Pressure | High Blood Pressure Diet Tips |
| Body Blood Pressure Calculator Prank | How to Reduce Blood Pressure |
| Blood Pressure Companion Pro | Blood Pressure Diary: BP Logger Scan Test Tracker |
| Blood Pressure BP Tracker for Hypertension Management | Treat High Blood Pressure Naturally |
| BloodPressureDB | 10 Yoga Poses High Blood Pressure |
| **Schizophrenia** | |
| Schizophrenia HealthStorylines | Schizophrenia Storylines |
| Schizophrenia | Remedies for Schizophrenia |
| Hearing Voices a Guide to Understanding Helping and Empowering Individuals | Schizophrenia Test |
| Schizophrenia Test | Schizophrenia: Causes, Diagnosis, and Management |
| 3D Brain | Understanding Schizophrenia |
| Psychiatry - Understanding Disease | Schizophrenia Info |
| Mental Health VR | Recognize Schizophrenia Disorder |
| Nearpod | Schizophrenia Test |
| ClinTouch | Schizophrenia |
| MHRG: Mental Health Recovery Guide | Schizophrenia |
